# Temporomandibular disorder and generalized joint hypermobility: application of diagnostic criteria

**DOI:** 10.1590/S1808-86942011000400003

**Published:** 2015-10-19

**Authors:** Fernanda Pasinato, Juliana Alves Souza, Eliane Castilhos Rodrigues Corrêa, Ana Maria Toniolo da Silva

**Affiliations:** 1 MSc in Human Communication Disorders - UFSM, Physical Therapist; 2 MSc in Human Communication Disorders - UFSM, Physical Therapist; 3 PhD in Oral-Dental biology - FOP/UNICAMP, Adjunct Professor - Department of Physical Therapy and Rehabilitation of the UFSM and Professor at the Graduate Program in Human Communication Disorders - UFSM; 4 PhD in Human Communication Disorders - UNIFESP, Adjunct Professor at the Department of Speech and Hearing Therapy and Professor at the Graduate Program in Human Communication Disorders - UFSM

**Keywords:** joint instability, temporomandibular joint disorders, evaluation

## Abstract

**Abstract:**

Generalized joint hypermobility (GJH) has been considered a predisposing factor for the development of temporomandibular disorder (TMD).

**Aim:**

To evaluate clinical and psychosocial aspects in individuals diagnosed with TMD with or without GJH.

**Materials and methods:**

Clinical and experimental study, which enrolled 34 women, from 18 to 35 years of age with TMD diagnosed by RDC/TMD. The GJH was assessed by the Beighton score and volunteers were broken down into 2 groups: with GJH (n = 22) and without GJH (n = 12).

**Results:**

We found a high percentage of GJH (64.71%). All participants had myofascial pain; 79.41% had arthralgia and 41% had disk displacement. There was a correlation between higher GJH scores and higher passive mouth opening amplitude (*p*=0.0034), with pain (*p*=0.0029) and without pain (*p*=0.0081). Greater mandibular range of motion was observed in the group with GJH, except for protrusion. Painful mouth opening was statistically higher in the GJH group (*p*=0.0279).

**Conclusions:**

Individuals with TMD associated or not to GJH do not differ significantly regarding clinical and psychosocial aspects, except in the mandibular opening range of motion, which if kept at physiological levels can lead to a late diagnosis of TMD in these individuals.

## INTRODUCTION

The term temporomandibular dysfunction has been used to define conditions involving changes to the structure and/or function of the masticatory system (TMJ and associated muscle-skeletal structures)^1,^^2^. The signs and symptoms which characterize this dysfunction are pain in the periauricular region, TMJ and masticatory muscles, noises, limitation or deviations during mandibular movement^3^.

Generalized joint hypermobility (GJH) is a hereditary characteristic defined by the increase in range of motion of multiple joints^4,^^5^. It can be understood as an isolate entity or making up the clinical condition of hereditary disorders of the connective tissue (syndromes)^6^.

Joint range of motion is influenced by numerous factors, including biochemical changes to the structure of collagen and elastin^7^, causing a loss of resistance to traction, laxity and increase in joint mobility.

GJH prevalence reported in the literature is much variable, it is known that gender, ethnicity and age are important factors. There are reports that this syndrome is more prevalent in women and in individuals of Asian and African descent, and it reduces with aging^8,^^9^.

GJH has been associated with the development of signs and symptoms of TMD^4^, ^5^, ^5^, ^6^,^10^, ^11^, ^12^, ^13^, ^14^, ^15^, ^16^. It is known that although the TMJ is among hypermobile joints, it may predispose some patients to subluxation, although not necessarily with pain or dysfunction^4^. Numerous studies^4,^^6,^^14,^^17^ investigated GJH repercussions on joint disorders, especially disc luxation. It is believed that because of ligament laxity, the joint is overloaded, resulting in degenerative changes which may manifest itself in internal derangements and joint inflammation^18^.

Some authors studied whether the mandibular opening range of motion could be indicative of hypermobility^19,^^20,^^5^. Studies have found associations between maximum mandibular opening and hypermobility^16,^^21^, while in others, this was not confirmed^19,^^20^.

One epidemiological study^16^ has shown that individuals with GJH have a higher risk of developing reciprocal click, indicating a diagnosis of disc luxation and a lower risk of mouth opening limitation. It has also reported a lower association between the diagnosis of myofascial pain and arthralgia (groups I and IIIa) and GJH, concluding that it seems to be associated to painless subtypes of TMD. The author considers that, with the increase in joint mobility in general, the greater is the maximum mouth opening, corroborating the hypothesis that the TMJ is involved in the GJH phenomenon. Consequently, a limitation in the opening movement may pass unnoticed because of the excess of TMJ mobility in these patients.

Bruxism is a parafunctional mouth habit characterized by pressing (concentric bruxism) or grinding the teeth (eccentric bruxism), being considered an etiological factor responsible for the beginning or perpetuation of the TMD^22,^^23^. According to Westling and Mattiasson^24^, this mouth parafunction seems to have a greater deleterious effect on people with joint hypermobility than their normal counterparts.

Other etiological factors found in individuals with TMD are depression and stress. Stress favors the discharge of tension on the chewing muscles, present in bruxism, and depression is frequently associated with chronic pain in these cases^25,^^26^. TMD diagnostic criteria (RDC/TMD) enable the assessment of these conditions by means of depression scores and unspecific physical symptoms, including pain or no pain, which represent physical manifestations of stress.

Individuals with GJH are also more commonly affected (69.3%) by stress disorders, when compared to groups of individuals with other rheumatic disorders (22%)^27^. The association between panic disorders and GJH has been explained by chromosome duplication, confirming the hereditariness of these conditions and its common biological etiology^28^.

As more health-care professionals and patients understand TMJ hypermobility, the more contributions we'll have to develop a more specific preventive approach to the TMD. Thus, the GJH could be included as a standard diagnostic component of this dysfunction.

There is no clear evidence of the association between TMD and GJH in the literature and this must be more intensely investigated, considering the small number of recent studies concerning the topic^18^.

Based on the above, the goal of the present study was to assess the clinical and psychosocial aspects of TMD in individuals diagnosed with and without general joint hypermobility.

## METHODS

The present study is a cross-sectional, observational study of quantitative and qualitative approach. It was developed at the Mouth Mobility Lab of the Speech and Hearing Therapy Service (SAF) of the school. The study was approved by the Ethics Committee of the Institution, under # 0281.0.243.000-08 and it was carried out between August of 2008 and June of 2009.

The sample was made up of 34 women diagnosed with TMD, with ages varying between 18 and 35 years. In the study we included individuals coming from the Occlusion Clinics Course of the School, or those who came to the researchers in response to the call in the printed and virtual media about the study, those who had one or more diagnosis of temporomandibular dysfunction, according to RDC/TMD^22^. All participants were informed of the nature and objectives of the study and they all signed the free and informed consent form.

The volunteers were broken down into two groups after the generalized joint hypermobility exam (Beighton's Score): Group without hypermobility (GN) and Group with hypermobility (GH).

We took off the study those volunteers with neuropsychomotor involvement, a past of orthopedic trauma or facial malformation; individuals with systemic or rheumatologic diseases, under physical therapy, dental or speech therapy current or previous, or those who were using any kind of medication.

The *Research Diagnostic Criteria* (RDC/TMD)^25^ is a method of data collection which is standard for the systematized diagnosis of TMD in two axes. Axis I of the RDC approaches the clinical aspects and classifies the TMDs as to diagnosis: Ia) myofascial pain; IIb) myofascial pain with restricted mouth opening; IIa) Disk luxation with reduction; IIb) Disk luxation without mouth opening restriction with reduction; IIc) Disc luxation without reduction and without opening reduction; IIIa) Arthralgia; IIIb) TMJ Osteoarthritis and IIIc) TMJ osteoarthrosis.

Individuals may be classified with no diagnosis or a maximum of five diagnoses. Among the patient's subjective findings we include the complaint of concentric and/or eccentric bruxism. Axis II assesses the psychosocial factors, enabling classification as to the chronic pain disability or intensity associated with the TMJ, besides the assessment of depression and non-specific physical symptoms.

The diagnosis of myofascial pain was based on the presence of self-referred pain upon rest and palpation sensitivity of three or more muscles in given places. Mouth opening was assessed by measuring the distance between the lower and superior incisive teeth by means of a digital caliper, and movement restraint was considered when mouth opening was ≤ 40mm. We also assessed the presence of joint deviation and click. Disc disorders were diagnosed in the presence of reproducible joint click. Opening limitations without joint noises characterized the diagnosis of disc luxation without reduction and the diagnosis of arthralgia and/or osteoarthritis/osteoarthrosis was confirmed in the presence of pain or joint crackling sounds.

RDC/TMD's 2nd axis enables one to classify the degree of chronic pain, depression and unspecific physical symptoms, based on a self-applicable questionnaire.

**Figure 1:**
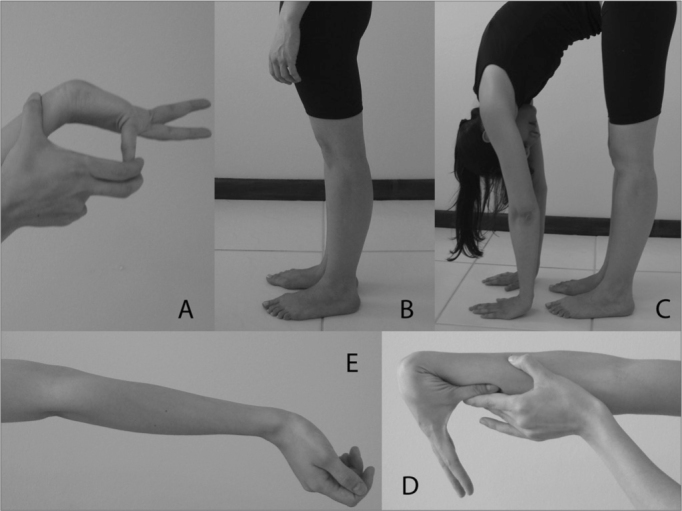
Assessment of generalized joint hypermobility (Beighton's Score). A) Little finger extension; B) Knee hyperextension; C) Trunk flexion with the palms of the hands touching the floor; D) Thumb flexion towards the forearm; E) Elbow hyperextension.

The GJH was assessed by the Carter and Wilkinson's criteria, modified by Beighton^29^, which have been used in numerous studies^4,^^6^,^11^, ^12^, ^13^, ^14^, ^15^, ^16^ about hypermobility. Beighton's score assesses 9 joints by means of 5 tests: passive thumb opposition to the anterior portion of the forearm, until they touch; passive dorsiflexion of the little finger until it comes parallel to the forearm; elbow and knee hyperextension higher than 10 degrees; trunk flexion with the knees completely extended, in such a way that the palms of the hands touch the floor (Figure 1).

Each joint with a positive test for GJH is scored. GJH is diagnosed when there is a score equal to or higher than 4, and it is ruled out in the presence of rheumatic diseases.

The *Mann-Whitney* (U Test) test was used in order to compare the mandibular range of motion mean values between the groups. We also used the chi-square and the Fisher's Exact test to compare the frequencies between the groups and the *Spearman* test to correlate mandibular movement range of motion and GJH scores.

## RESULTS

Individuals with TMD were classified as to the presence of generalized joint hypermobility (GJH) according to the Beighton's Score. 64.71% of the individuals evaluated had scores equal to or higher than 4, and they were classified as having GJH. Of these, 41.18% had moderate GJH (4-6 points) and 23.53% severe (7-9 points). Normal joint mobility was seen in 35.29% of the individuals with TMD. The mean, standard deviation and statistical significance level (U test) of the mandible range of motion measures from individuals with TMD with and without GJH are depicted on Table 1.

**Table 1:** Mean, standard deviation and significance level of the mandible range of motion measures of individuals with TMD distributed as to the presence of GJH.

	GN		GH		U Test
Mandibular ROM	Mean	SD	Mean	SD	*p*
Painless opening	37.43	7.02	41.75	8.20	0.0903
Painful opening	45.95	4.11	51.20	7.04	0.0279*
Passive opening	49.35	3.04	53.46	6.64	0.0774
Right lateral shift	8.89	3.05	10.05	2.14	0.2638
Left lateral shift	8.58	2.93	9.63	3.14	0.3305
Protrusion	4.81	2.35	4.61	1.13	0.6785

Acronyms: GN= Group without GJH; GH= Group with GJH; SD= Standard Deviation *statistical significance (*p*< 0.05).

There were higher ranges of motion in the GJH, except for protrusion. In the mouth opening with pain measure, this group had a statistically higher value when compared to the group without hypermobility.

Mandibular range of motion amplitude values were correlated to the GJH scores using the *Spearman test*, and we found a positive correlation between the active and painful mandibular opening range of motion (r=0.49 and *p*= 0.0029), painless (r=0.45 and *p*=0.0081) and upon passive opening (r=0.49 and *p*=0.0034) and the GJH score. There was no correlation on the right and left laterotrusion analysis and in protrusion.

The results from the palpation of the masseter and temporal muscles, lateral pole and posterior ligament are listed on Table 2. The group of patients without GJH had higher percentages of muscle and joint severe pain when compared to the hypermobility group. Nonetheless, the statistical difference was not confirmed by the chi-square test.

**Table 2:** Results from the muscle and joint palpation of individuals with TMD distributed according to the presence of GJH.

	Classificação da dor	GN (n=12)		GH (n=22)		Chi-square Fisher
	Masseter	f	%	f	%	*P*
	Sem dor	0	0	0	0	-
	Dor leve	0	0	2	9.09	-
	Dor moderada	1	8.33	4	18.18	NS
Muscle palpation	Dor severa	11	91.67	16	72.72	NS
	Temporal Anterior					
	Sem dor	0	0	4	18.18	-
	Dor leve	1	8.33	2	9.09	NS
	Dor moderada	5	41.67	8	36.36	NS
	Dor severa	6	50	8	36.36	NS
	Polo Lateral da ATM					
	Sem dor	0	0	1	4.54	-
	Dor leve	2	16.67	2	9.09	NS
	Dor moderada	4	33.33	6	27.27	NS
Joint palpation	Dor severa	6	50	13	59.02	NS
	Ligamento Posterior					
	Sem dor	2	16.67	7	31.82	NS
	Dor leve	2	16.67	3	13.64	NS
	Dor moderada	3	25	6	27.27	NS
	Dor severa	5	41.66	6	27.27	NS

Acronyms: GN= Group without GJH; GH= Group with GJH; f = frequency; NS= Not significant.

All individuals with TMD were diagnosed with myofascial pain (Group I), 41% were diagnosed with disc disorders (Group II) and 91% had some type of joint involvement (Group III), especially arthralgia (79.41%) (Table 3).

**Table 3:** Percentage results of the TMD diagnostic classification according to the RDC/TMD criterion (Dworkin & LeResche, 1992), distributed as to the presence of GJH.

Classification		TMD	GN (n=12)	GH (n=22)	Chi-square Fisher
		%	%	%	*p*
GROUP I	Ia	70.59	58.33	81.82	NS
	Ib	29.41	41.67	18.18	NS
	None	0	0	0	-
	IIa	38.23	41.67	31.82	NS
GROUP II	IIb	0	0	0	-
	IIc	2.94	0	4.54	-
	None	61.76	58.33	63.64	NS
	IIIa	79.41	83.33	81.82	NS
GROUP III	IIIb	11.76	8.33	9.09	NS
	IIIc	2.94	0	4.54	-
		8.82	8.33	9.09	NS

Acronyms: Ia= myofascial pain; Ib= myofascial pain with restrained opening, IIa=disc luxation with reduction; IIb= disc luxation without reduction with restrained opening; IIc= disc luxation without reduction without restrained opening; IIIa= arthralgia; IIIb= TMJ osteoarthritis; IIIc= TMJ osteoarthrosis; GN= Group without GJH; GH= Group with GJH. NS= Non-significant.

When participants were broken down according with the presence of GJH, we noticed a higher percentage of myofascial pain without mouth opening restraints (Ia) in individuals with GJH (81.82%) when compared to the group without GJH (58.33%). This difference was not significant in the chi-square test (*p*= 0.2468).

Disc dislocation with reduction (IIa) was diagnosed in 31.82% of hypermobile individuals and in 41.67% of the participants with normal joint hypermobility. The diagnosis of arthralgia (IIIa) was high in both groups (81.82 and 83.33% in the groups with and without GJH, respectively).

The classification of chronic pain, depression and non-specific physical symptoms, including pain or no pain, are depicted on Table 4. The participants were classified according to the axis II of the RDC as to the degree of chronic pain: grade I (low disability and low intensity), grade II (low disability and high intensity), grade III (high disability and moderate limitation) and grade IV (high disability and severe limitation). Among individuals with normal joint mobility, 58.34% were diagnosed with chronic pain grades II and III. When GJH was present, this value was 36.36%. We employed the chi-square test and it did not yield statistical difference in the incidence of chronic pain among the groups assessed (*p* = 0.412).

**Table 4:** Frequency distribution (%) of the classification of chronic pain, depression and non-specific physical symptoms, including or excluding pain items (RDC/TMD) in individuals with TMD with and without GJH.

	GEN (n=12)		GEH (n=22)		Qui-Quadrado Fischer
Chronic Pain Classification	f	%	f	%	*p*
Grade I	5	41.67	14	63.64	NS
Grade II	5	41.67	6	27.27	NS
Grade III	2	16.67	2	9.09	NS
Depression					
Normal	2	16.66	1	4.54	NS
Moderate	7	58.34	13	59.1	NS
Severe	3	25	8	36.36	NS
Physical symptoms including pain					
Normal	1	8.33	2	9.09	NS
Moderate	4	33.33	7	31.82	NS
Severe	7	58.34	13	59.09	NS
Physical symptoms excluding pain					
Normal	3	25	5	22.73	NS
Moderate	2	16.66	7	31.82	NS
Severe	7	58.34	10	45.45	NS

Acronyms: GN= Group without GJH; GH= Group with GJH; f = frequency; NS=not significant

The percentage values of moderate and severe depression were similar between the groups, and the higher presence of severe depression was found among those individuals with GJH. As to the classification of non-specific physical symptoms, including pain or not, there were no percentage differences between the groups.

The occurrence of concentric and eccentric bruxism was reported by a large number of individuals, in GN 66.67% and 91.67% reported these disorders, respectively. In GH, 72.73% had concentric bruxism and 90.91% had the eccentric type.

## DISCUSSION

Temporomandibular disorders are usually described as multifactorial disorders. Generalized joint hypermobility has been reported as a predisposing factor to the deve- lopment of TMD, and some studies^6,^^11,^^14^ found a higher incidence of this trait among individuals with signs and symptoms of TMD. In the present study we noticed a high percentage of individuals with GJH (64.71%), in agreement with Kavuncu et al.^14^, who found 79.7% of GJH in individuals with TMD.

The age range of participants was, in average, 25 years of age. The incidence of GJH reduces as age increases^4,^^29^; thus, the young age of the participants also justifies its high incidence in the group assessed.

We found mean values of mandibular range of motion within the normal parameters reported in the literature^30^, except for the movement of protrusion, which mean value observed was 4.81 for GN and 4.61 for GH, below reference levels (≥ 7mm). Concerning the opening with pain, the group without hypermobility presented a mean value of 37.43mm, which was also below normal values (≥ 40mm).

Higher mandibular range of motion values were found in the hypermobile group, and a statistically higher value was found in painful mouth opening. We noticed a positive moderate correlation between higher GJH scores and a greater passive and active mouth opening range of motion with and without pain. Winocur^5^ also found a positive correlation between GJH and maximum mouth opening in hypermobile individuals. Salomão and Barbosa^31^ added that this correlation must be considered in the evaluation of hypermobile patients, because in this, a 45mm clinical finding of opening, although within normal standards may mean movement restriction.

On the other hand, Perrini et al.^11^, despite finding an association between TMD and GJH, did not find greater mandibular range of motion in hypermobile individuals. Westling and Helkimo^20^ also did not find any association between maximum mouth opening and greater mobility of peripheral joints in asymptomatic individuals.

In an epidemiological study on TMD risk factors^16^ the authors found that hypermobile individuals had lower risk of having limited mouth opening. Nonetheless, in numerous studies^4,^^11,^^20^ the relationship between GJH, increase in mouth opening range of motion (condylar hypertranslation) and TMD was not confirmed. Kavuncu et al.^14^ assessed the risk of temporomandibular dysfunction when the patient has GJH and condylar hypermobility and noticed that these were more frequent in individuals with TMD, and that the risk of the individual developing this disorder is higher when they happen simultaneously. In the present study condylar hypermobility was not assessed.

In a longitudinal study, Dijkstra et al.^32^ also did not notice clinical and functional differences between individuals with and without TMD hypermobility, nonetheless, they had higher incidences of radiographic signs of osteoarthrosis. These signs, without clinical manifestations and functional loss may lead to a diagnosis of advanced TMD. This justifies the importance of an early investigation of TMD in hypermobile individuals.

According to the RDC/TMD, multiple disorders were present in most of the individuals with TMD, and they all had myofascial pain; 91.12% had some joint involvement, especially arthralgia (79.41%). Disc disorders were present in 41% of the individuals. Suvinen et al.^33^ found similar results, with a prevalence of mixed myogenic and arthrogenic dysfunction in 44% of the individuals. Nonetheless, in studies involving older individuals^34,^^35^, the diagnosis of disc disorders was more frequent, and there are reports^11^ that disc luxation diagnosis increases with age. In comparing TMD individuals with and without GJH, the percentage values were similar considering the diagnosis of arthralgia and myofascial pain.

A higher percentage of myofascial pain without mouth opening restrictions was found in individuals with GJH (81.82%) when compared to the group without GJH (58.33%); however, this difference was not significant. Hirsh et al.^16^ confirmed a lower risk of individuals with GJH develop mouth opening restrictions. The preservation of mandibular range of motion within biological parameters, in these individuals, may lead to a low functional repercussion and late diagnosis.

The prevalence of disc disorder was similar in both groups (31.82% and 41.67% in the groups with and without hypermobility, respectively). This is in agreement with the findings by Conti et al.^4^ and Saéz-Yuguero et al.^17^. In their study, Perrini et al.^11^ did not report a higher prevalence of GJH in individuals with bilateral disc luxation. On the other hand, other studies^6,^^14^ found the association between hypermobility and this type of dysfunction.

Hisch et al.^16^ decided to analyze whether GJH is a risk factor for TMD in a study involving 895 individuals and noticed an association between GJH, increase in mandibular range of motion and reciprocal *click*. Our study has confirmed these findings, except for the *click.*

In the present study, most of the participants reported concentric or eccentric bruxism which, when associated with hypermobility, may play a role in the patient's developing and maintaining TMD. Westling and Mattiasson^24^ stated that the mouth parafunction seems to have a greater deleterious effect on those individuals with joint hypermobility. According to these authors, there is no evidence that mouth parafunction would cause TMD; nonetheless, a systemic factor such as GJH may play an important role when the masticatory system is exposed to overloads as it happens during parafunction.

On the other hand, Boering apud Winocur et al.^5^ stated that hypermobility by itself does not affect the TMJ, unless there is a movement dysfunction such as a *click* or a joint lock. Thus, the GJH-associated parafunction may be worse to the TMJ, since the condylar translation movement happens with a higher pressure because of the joint overload.

We did not find statistically significant differences on the incidence of chronic pain and muscle palpation and joint pain between the groups. Nonetheless, the GJH group had lower incidences of severe muscle and joint pain, and fewer grades II and III of chronic pain (36.36% *versus* 58.34% in the group without GJH). These results may indicate that the clinical manifestations of individuals with GJH are less evident. Winocur et al.^5^ did not notice statistically significant associations between TMJ hypermobility and signs and symptoms of TMD, which included mandible range of motion, presence of *clicks* and sensitivity in muscle and joint palpation.

High percentages of depression and non-specific symptoms were noticed in the individuals assessed; nonetheless, we did not find statistically significant differences when the individuals were compared as to the presence of GJH.

Depression is the emotional state which is most commonly associated with chronic pain; notwithstanding, stress may also be associated with TMD. Individuals with TMD are more stressed and/or depressed than asymptomatic individuals, and TMD symptoms start during periods of psychological stress and flare up during stressful situations^36^, ^37^, ^38^.

## CONCLUSION

Generalized joint hypermobility was highly prevalent in individuals with TMD. Considering the general analysis of the results obtained from the groups studied, we concluded that individuals with TMD associated or not with generalized joint hypermobility do not differ as to the clinical and psychosocial aspects assessed, except concerning the mandibular opening range of motion.

The possible clinical implication of this result is that individuals with TMD associated with GJH may maintain TMJ range of motion within physiological limits, even upon its reduction, which could cause the late diagnosis of this condition.

Thus, patient awareness concerning this condition may prevent lesions caused by actions or situations which require excessive mandibular opening (yawning, broad mouth opening during feeding, long dental procedures), acting preventively.
